# Clinical Efficacy and *In Vitro* Drug Sensitivity Test Results of Azithromycin Combined With Other Antimicrobial Therapies in the Treatment of MDR *P. aeruginosa* Ventilator-Associated Pneumonia

**DOI:** 10.3389/fphar.2022.944965

**Published:** 2022-08-10

**Authors:** Yuqin Huang, Wenguo Wang, Qiang Huang, Zhengyan Wang, Zhuanzhuan Xu, Chaochao Tu, Dongli Wan, Miaobo He, Xiaoyi Yang, Huaqiang Xu, Hanqin Wang, Ying Zhao, Mingli Tu, Quan Zhou

**Affiliations:** ^1^ Intensive Care Unit, Suizhou Central Hospital, Hubei University of Medicine, Suizhou, China; ^2^ Department of Respiratory Medicine, Suizhou Central Hospital, Hubei University of Medicine, Suizhou, China; ^3^ Department of Medicine, First Clinical School, Guangzhou Medical University, Guangzhou, China; ^4^ Center for Translational Medicine, Suizhou Central Hospital, Hubei University of Medicine, Suizhou, China; ^5^ Department of Clinical Laboratory, Taihe Hospital, Hubei University of Medicine, Shiyan, China

**Keywords:** ventilator-associated pneumonia, *Pseudomonas aeruginosa*, azithromycin, *in vitro* drug sensitivity test, multidrug-resistant

## Abstract

**Objective:** The aim of the research was to study the effect of azithromycin (AZM) in the treatment of MDR *P. aeruginosa* VAP combined with other antimicrobial therapies.

**Methods:** The clinical outcomes were retrospectively collected and analyzed to elucidate the efficacy of different combinations involving azithromycin in the treatment of MDR-PA VAP. The minimal inhibitory concentration (MIC) of five drugs was measured by the agar dilution method against 27 isolates of MDR-PA, alone or in combination.

**Results:** The incidence of VAP has increased approximately to 10.4% (961/9245) in 5 years and 18.4% (177/961) caused by *P. aeruginosa* ranking fourth. A total of 151 cases of MDR *P. aeruginosa* were included in the clinical retrospective study. Clinical efficacy results are as follows: meropenem + azithromycin (MEM + AZM) was 69.2% (9/13), cefoperazone/sulbactam + azithromycin (SCF + AZM) was 60% (6/10), and the combination of three drugs containing AZM was 69.2% (9/13). The curative effect of meropenem + amikacin (MEM + AMK) was better than that of the meropenem + levofloxacin (MEM + LEV) group, *p* = 0.029 (*p* < 0.05). The curative effect of cefoperazone/sulbactam + amikacin (SCF + AMK) was better than that of the cefoperazone/sulbactam + levofloxacin (SCF + LEV) group, *p* = 0.025 (*p* < 0.05). There was no significant difference between combinations of two or three drugs containing AZM, *p* > 0.05 (*p* = 0.806). From the MIC results, the AMK single drug was already very sensitive to the selected strains. When MEM or SCF was combined with AZM, the sensitivity of them to strains can be significantly increased. When combined with MEM and AZM, the MIC_50_ and MIC_90_ of MEM decreased to 1 and 2 ug/mL from 8 to 32 ug/mL. When combined with SCF + AZM, the MIC_50_ of SCF decreased to 16 ug/mL, and the curve shifted obviously. However, for the combination of SCF + LEV + AZM, MIC_50_ and MIC_90_ could not achieve substantive changes. From the FIC index results, the main actions of MEM + AZM were additive effects, accounting for 72%; for the combination of SCF + AZM, the additive effect was 40%. The combination of AMK or LEV with AZM mainly showed unrelated effects, and the combination of three drugs could not improve the positive correlation between LEV and AZM.

**Conclusion:** AZM may increase the effect of MEM or SCF against MDR *P. aeruginosa* VAP. Based on MEM or SCF combined with AMK or AZM, we can achieve a good effect in the treatment of MDR *P. aeruginosa* VAP.

## Introduction

Ventilator-associated pneumonia (VAP) refers to the pneumonia that occurs after endotracheal intubation or tracheotomy patients receiving mechanical ventilation (MV) for 48 h. The pneumonia that occurs within 48 h after MV withdrawal and extubation also belongs to the category of VAP (Kalil et al., 2016; Torres et al., 2017; Shi et al., 2019). VAP is a common nosocomial infection in critically ill patients. With the extensive application of invasive MV in the rescue of intensive care unit (ICU) patients, VAP has become one of the most common complications, presenting high incidence rate and mortality (Shi et al., 2019). Statistical results showed that the incidence rate of VAP was 7.9%–48.4%, and the mortality was 21.2%–43.2% (Metersky and Kalil, 2018; Papazian et al., 2020). Once patients are combined with VAP, the time of MV, the length of hospital stay, and the cost of hospitalization will increase, and some cases are even life-threatening. All of these directly affect the short-term and long-term prognosis of patients (Kalanuria et al., 2014). Gram-negative flora is the majority in the VAP pathogen spectrum, including *Acinetobacter baumannii*, *P. aeruginosa*, *Klebsiella pneumoniae*, *Escherichia coli*, and so on (Kalil et al., 2016). *P. aeruginosa* is widely recognized as a common conditional pathogen of hospital-acquired infection (Faure et al., 2018). *P. aeruginosa* has the characteristics of easy colonization, variation, and multi-drug resistance (MDR) (Miyoshi-Akiyama et al., 2017; Liao et al., 2019; Al-Orphaly et al., 2021). Among Gram-negative strains, the most common MDR pathogens are *Acinetobacter baumannii*, *P. aeruginosa*, and Enterobacteriaceae (Otsuka, 2020; Mills and Marchaim, 2021). In China, carbapenem-resistant *Pseudomonas aeruginosa* (CR-PA) has been included in one of the five MDR bacteria targeted for prevention and control, in accordance with the requirements of the National Health Commission. The US Centers for Disease Control and Prevention antibiotic resistance threat report has stated that three groups of antimicrobial resistant Gram-negative bacteria pose particular therapeutic challenges: 1) extended-spectrum β-lactamase producing Enterobacterales (ESBL-E), 2) carbapenem-resistant Enterobacterales (CRE), and 3) *Pseudomonas aeruginosa* with difficult-to-treat resistance (DTR *P. aeruginosa*). These pathogens have been designated urgent or serious threats by the CDC in the United States.

MDR and pan-drug resistant (PDR) strains of *P. aeruginosa* are particularly frequent in ICU-acquired pneumonia (Ribeiro et al., 2019; Souza et al., 2021). The isolation of a MDR pathogen has been identified as an independent predictor of increased mortality in VAP. In recent years, with the abuse of broad-spectrum antibiotics, MDR and PDR *P. aeruginosa* have been increasing, which bring great difficulties to clinical treatment. The mechanism of *P. aeruginosa* resistance is complex, especially the formation of biofilm (Maurice et al., 2018), which leads to strong bacterial resistance at the lesion. Biofilm formation is the main reason for the recurrence and difficulty to control disease after *P. aeruginosa* infection.

In the past few years, the combination of the two drugs has been frequently used to treat MDR *P. aeruginosa* VAP in the ICU (Shi et al., 2019). The combined antibacterial scheme was based on carbapenems or cephalosporins β-lactamase inhibitor combinations, combined with fluoroquinolones or aminoglycosides, in accordance with the recommendations of the guidelines (Kalil et al., 2016; Torres et al., 2017; Shi et al., 2019). Among these types of drugs, the most commonly used drugs are: meropenem, cefoperazone sulbactam, levofloxacin, and amikacin. In the past, there was no polymyxin in our hospital. For PDR *P. aeruginosa* VAP, our treatment was very difficult. However, we found that a regimen of azithromycin combined with the aforementioned drugs may improve the symptoms of patients and achieve good therapeutic results. However, azithromycin is not the drug recommended in the guidelines for the treatment of *P. aeruginosa* pneumonia. Even *P. aeruginosa* is naturally resistant to azithromycin. So, when it is used in combination, how does it play an antibacterial role? Therefore, this study aimed to research the efficacy of azithromycin combined with other treatment regimens in the treatment of MDR-PA VAP through retrospective analysis of clinical data and *in vitro* drug sensitivity tests.

In this study, 5 years of clinical data from January 2017 to December 2021 were studied retrospectively to describe the characteristics of PA-VAP, and determine the clinical efficacy of antimicrobial regimens. A total of 27 strains of MDR-PA were isolated from our ICU from June 2021 to February 2022. According to the principle of clinical medication, five antibiotics, namely, meropenem (MEM), cefoperazone sulbactam (SCF), amikacin (AMK), levofloxacin (LEV), and azithromycin (AZM), as single drug or combination, were used for the *in vitro* drug sensitivity test to provide the evidence for the clinical treatment. It aimed to study the efficacy and mechanism of azithromycin combined with other regimens in the treatment of MDR *P. aeruginosa* VAP by combining the results of clinical analysis and *in vitro* drug sensitivity test.

## Materials and Methods

### Setting and Study Design

A retrospective study was conducted in the general ICU of Suizhou Central Hospital Affiliated to Hubei University of Medicine from 1 January 2017 to 31 December 2021. Suizhou Central Hospital is a 2380-bed tertiary care comprehensive hospital, which receives about 73,300 admissions per year. The ICU has 52 beds and covers all medical and surgical cases. This study was approved by the Ethics Committee of Suizhou Central Hospital.

The study included all adult patients who were mechanically ventilated for >48 h and developed VAP caused by *P. aeruginosa*. The first episode of *P. aeruginosa* VAP or polymicrobial VAP was recorded for each patient. The patients with other previous or concurrent infections were excluded from the study. Eligible patients were recognized by the microbial culture results to identify MDR isolates. The patients with COVID-19 were not included in this study. (Special management requirements based on the hospital, since the occurrence of novel coronavirus pneumonia, we have always had a special isolation ward to treat patients with severe COVID-19 pneumonia).

### Definitions

VAP was defined according to the guidelines of Chinese Thoracic Society (CTS) and the ATS-IDSA (Kalil et al., 2016; Shi et al., 2019). Diagnosis of VAP required radiographic appearance of a new or persistent pulmonary infiltrate and two or more of the following criteria:

① Temperature of >38°C or <36°C.
② Leukocytosis (peripheral blood leukocyte count, >10×10^9^/L) or leukopenia (peripheral blood leukocyte count, <4×10^9^/L).
③ The presence of purulent bronchial secretions.

Pneumonia was considered to be ventilator-associated when onset occurred 48 h after the initiation of MV, and was judged not to have been incubating before the initiation of MV. The patients with no clinical symptoms or radiological evidence of an infiltrate were excluded from the study. The onset of VAP was defined as the date of collection of the first clinical positive microbial cultures of aspirate:

① Specimen cultures obtained by endotracheal aspiration cultures (ETA) >10^5^ CFU/ml; or ② bronchoalveolar lavage cultures (BAL) >10^4^ CFU/ml.

MDR pathogens were commonly resistant to at least three classes of the following five antibiotics: cephalosporins, carbapenems, compound preparation containing β-lactamase inhibitor, fluoroquinolone, and aminoglycoside antibiotics. Clinical pulmonary infection scores (CPIS) were a retrospective calculation for the studied cases given the nature of this study.

### Empirical Antimicrobial Agents’ Plan and Curative Effect Judgment

The usage and dosage of each antibacterial drug are as follows:

Meropenem, 1g pump in for 3 h, every 8 h.
Cefoperazone/sulbactam, 3 g intravenous drip, every 12 h.
Amikacin, 15 mg/kg, intravenous drip, once a day.
Levofloxacin, 0.4 g intravenous drip, once a day.
Azithromycin, 0.5 g intravenous drip, once a day.

All patients involved in the study received appropriate antibiotic therapy. The course of all drug combination treatments was for at least 7–10 days. Experiential treatment schemes are as follows:

① Meropenem + amikacin.
② Meropenem + levofloxacin.
③ Cefoperazone/sulbactam + amikacin.
④ Cefoperazone/sulbactam + levofloxacin.
⑤ Meropenem or cefoperazone/sulbactam + levofloxacin or amikacin + azithromycin.

The clinical outcome of PA-VAP was a comprehensive judgment based on the clinical symptoms and CPIS of the patients.

Cured: the clinical symptoms were eliminated, and the results of sputum culture turned negative.

Improved: the clinical symptoms were obviously improved, and the CPIS was declined before combination therapy.

Aggravated: the clinical symptoms were worse, and the CPIS was increased before combination therapy.

Dead: VAP-related death was defined as death that occurred during the treatment period when the signs of pneumonia remained, or due to septic shock.

Effective treatment cases = cured + improved cases.
Ineffective treatment cases = aggravated + dead cases.

### Clinical Data Collection

Clinical, biological, and treatment data were obtained retrospectively from patient medical records and department of nosocomial infection management databases. Clinical data included age, sex, Acute Physiology and Chronic Health Evaluation (APACHE) II scores, ICU admission diagnosis, comorbidities, days of MV to VAP, as well as possible risk factors for MDR.

Drug sensitivity data of *P. aeruginosa* to 14 antibiotics from 2017 to 2021 were collected for analysis of drug resistance rate and trend. Data on antimicrobial therapy for the group of *P. aeruginosa* VAP were recorded for assessment of the effectiveness. The cases were grouped according to the different treatment schemes mentioned earlier. Clinical outcomes were analyzed to elucidate the effect of these empiric antibiotic regimens.

### Combined Drug Sensitivity Test *In Vitro*

A total of 27 strains of MDR *P. aeruginosa* were isolated from different patients in the ICU of our hospital in June 2021–February 2022. Quality control strains: *Pseudomonas aeruginosa* ATCC 27853. The minimum inhibitory concentration (MIC) of a single drug was determined in accordance with the method recommended by CLSI (M100ED32-2022) (Clinical and Laboratory Standards Institute, 2022). The MIC value of meropenem, cefoperazone/sulbactam, amikacin, levofloxacin, and azithromycin against 27 strains of *P. aeruginosa* was determined using the agar dilution method. Mueller–Hinton (MH) broth was diluted to a series of concentrations by a double ratio, and all of the five antibiotics were diluted to 11 concentration gradients. The concentrations in the combined drug sensitivity test are 512, 256, 128, 64, 32, 16, 8, 4, 2, 1, and 0.5 (ug/mL). The specific experimental steps are as follows: ①preparation of the culture medium: MH agar was used to prepare the culture medium according to requirements; ② preparation of agar plate containing drugs: add the diluted antibacterial drugs of different concentrations into the quantitative MH agar melted and cooled to about 50°C, and the plate containing antibacterial drugs of different decreasing concentrations was made. Put it in a sealed plastic bag and store it in a refrigerator at 2–8°C for 5 days; ③ inoculation: inoculate the bacterial solution on the surface of the agar plate, incubate at 35°C for 16–20 h after inoculation; ④ result judgment: place the plate on the surface of dark and non-reflective objects to judge the test end point, and the minimum drug concentration contained in the agar plate that inhibits bacterial growth was regarded as MIC. The single drug MIC (MIC_A alone_ and MIC_B alone_) and the MIC value of the optimal combination effect (MIC_A combined_ and MIC_B combined_) were selected to record. The combined drug sensitivity test usually uses the fractional inhibitory concentration (FIC) value to evaluate the effect of the combined drug use. The calculation method and criterion of interpretation of the FIC index are: FIC index = MIC_A combined_/MIC_A alone_ + MIC_B combined_/MIC_B alone_, synergistic: FIC ≤ 0.5, addictive: 0.5 < FIC ≤ 1, indifference: 1 < FIC ≤ 2, and antagonistic: FIC > 2.

### Statistical Analysis

SPSS 24.0 and Excel software were used for statistical analysis, *p* < 0.05 was found to be statistically significant. Qualitative variables were expressed as percentages, whereas quantitative variables are expressed as means ± standard deviations (SD) or medians.

## Result

Between January 2017 and December 2021, 10,272 adult patients were admitted to our ICU and 9,245 cases were mechanically ventilated patients. The diagnostic criteria for VAP were fulfilled in 961 patients (10.4%, 961/9245), 177 episodes of VAP were due to *P. aeruginosa*, and the incidence of PA-VAP has approximately 18.4% (177/961) of all VAP patients, ranked fourth (*Acinetobacter baumannii*, *Escherichia* coli, and *Klebsiella pneumoniae* ranked in the top three).

A total of 151 patients were included in our study and 26 patients were excluded from this analysis because VAP treatment time was not enough or took other plans for treatment. They were divided into seven groups based on the treatment regimen (Table.3 for details). We analyzed the drug sensitivity of five different combination regimens of antibiotics based on the 25 strains of MDR-PA from different patients in the ICU of our hospital in June 2021–February 2022. Note: 27 strains and one quality control strain were used for the *in vitro* drug sensitivity test. However, during the test, two strains were not successful and were excluded from the analysis of the results.

### Clinical Characteristics of 151 Patients Treated for PA-VAP

The mean age of the patients was 50.4 ± 11.3 years old (ranging from 25 to 86 years). The male to female ratio was 2.4 (males 106: females 45). The APACHE II score was 23 ± 5. In the hospital, 52 patients (34.4%) were admitted because of multiple trauma, 49 (32.5%) were admitted because of severe craniocerebral trauma, and 30 (19.9%) were admitted for severe nervous system disease. Also, 28 patients (18.5%) had a previous history of hypertension, 16 patients (10.6%) had a previous history of chronic obstructive pulmonary disease (COPD), 14 patients (9.3%) had other respiratory diseases, diabetes mellitus was known for 11 patients (7.3%), coronary heart disease was known for 9 patients (6.0%), and digestive system disease was known for 11 patients (7.3%). The mean length of hospital admission to VAP was 8.4 ± 5.3 days, the mean length of ICU admission to VAP was 7.5 ± 4.6 days, and the mean time from MV to VAP was 6.8 ± 3.4 days. The duration of MV was 13.4 ± 11.4 days, the length of ICU stay was 20.3 ± 7.8 days, and the length of hospital stay was 31.2 ± 11.5 days. The clinical characteristics and outcomes of all patients are summarized in Table 1.

**TABLE 1:** Clinical characteristics of patients treated for PA-VAP (*n* = 151).

Characteristic	
No.	151
Age, mean ± SD (years)	50.4 ± 11.3
Female sex [n (%)]	45 (29.8)
Male sex [n (%)]	106 (70.2)
APACHE II score, mean ± SD	23 ± 5
ICU admission diagnosis [n (%)]	
Multiple trauma	52 (34.4)
Severe craniocerebral trauma	49 (32.5)
Respiratory failure	3 (23.8)
Severe nervous system disease	30 (19.9)
Hemorrhagic shock	16 (10.6)
Various kinds of poisoning	13 (8.6)
Acute exacerbation of COPD	10 (6.6)
Other reasons	12 (7.9)
Comorbidities [n (%)]	
Hypertension	28 (18.5)
COPD	16 (10.6)
Other respiratory diseases	14 (9.3)
Diabetes	11 (7.3)
Coronary heart disease	9 (6.0)
Digestive system disease	11 (7.3)
None	68 (45)
Days of hospital admission to VAP, mean ± SD	8.4 ± 5.3
Days of ICU admission to VAP, mean ± SD	7.5 ± 4.6
Days of MV to VAP, mean ± SD	6.8 ± 3.4
Early-onset VAP [n (%)]	24 (15.9)
Late-onset VAP [n (%)]	127 (84.1)
Duration of MV, days, mean ± SD	13.4 ± 11.4
Length of ICU stay, days, mean ± SD	20.3 ± 7.8
Length of hospital stay, days, mean ± SD	31.2 ± 11.5

Abbreviations: APACHE, acute physiology and chronic health evaluation; ICU, intensive care unit; COPD, chronic obstructive pulmonary disease; MV, mechanical ventilation.

### Antimicrobial Resistance of *P. aeruginosa* of VAP From 2017 to 2021

In this retrospective study, 151 strains of *P. aeruginosa* were all MDR bacteria. In 5 years, the highest incidence is in 2019, up to 57 of these 151 cases, accounting for 37.75%.

**FIGURE 1:**
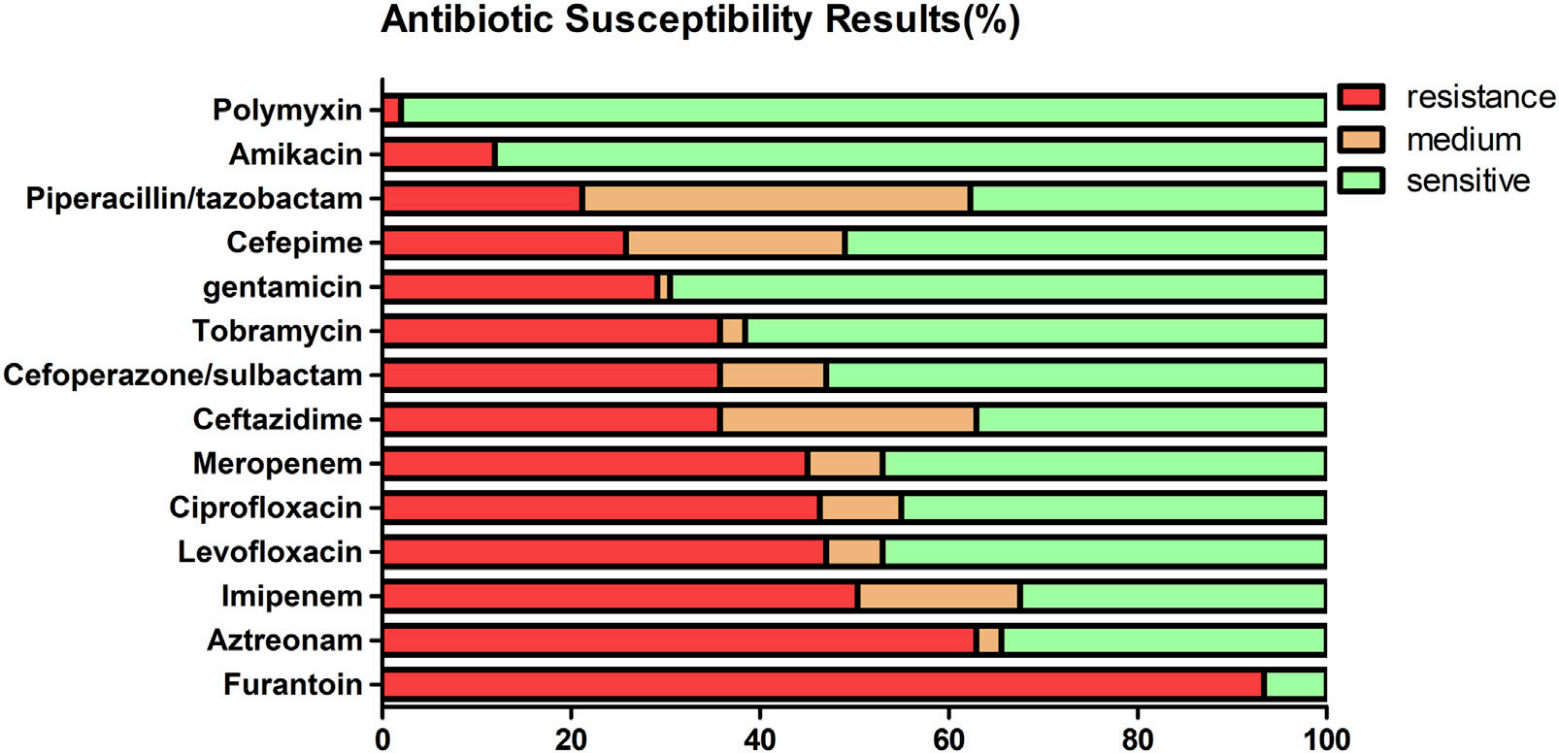
Antimicrobial resistance of the 151 isolates of *P. aeruginosa* to 14 antibiotics from 2017 to 2021 (%).

Table 2 and Figure 1 show the sensitivity and resistance of *P. aeruginosa* to 14 antibiotics in 5 years. Among the 14 antibiotics, the three most sensitive are polymyxin (98%), amikacin (88.1%), and gentamicin (69.5%). Accordingly, the three most resistant are furantoin (93.4%), aztreonam (62.9%), and imipenem (50.3%), the resistance rates were over 50%. The resistance rates of *P. aeruginosa* to meropenem, cefoperazone/sulbactam, amikacin, and levofloxacin are as follows: 45.0%, 35.8%, 11.9%, and 47.0%, respectively. The details are shown in Table 2. It was worth mentioning that *P. aeruginosa* showed high resistance to carbapenem antibiotics from the clinical drug sensitivity results. The resistance rates of *P. aeruginosa* to imipenem and meropenem are 50.3% and 45.0%, respectively.

**TABLE 2:** Antimicrobial resistance of the 151 isolates of *P. aeruginosa* to 14 antibiotics from 2017 to 2021 (%).

Antibiotic	S	R	I	Sensitivity rate (%)	Resistance rate(%)
Amikacin	133	18	0	88.1	11.9
Ceftazidime	56	54	41	37.1	35.8
Ciprofloxacin	68	70	13	45.0	46.4
Gentamicin	105	44	2	69.5	29.1
Furantoin	10	141	0	6.6	93.4
Cefepime	77	39	35	51.0	25.8
Imipenem	49	76	26	32.5	50.3
Levofloxacin	71	71	9	47.0	47.0
Tobramycin	93	54	4	61.6	35.8
Piperacillin/tazobactam	57	32	62	37.7	21.2
Aztreonam	52	95	4	34.4	62.9
Meropenem	71	68	12	47.0	45.0
Cefoperazone/sulbactam	80	54	17	53.0	35.8
Polymyxin	148	3	0	98.0	2.0

Abbreviations: S, susceptible; R, resistant; I, intermediate.

### Therapeutic Effect of the Combination of Five Antibiotics

Table 3 shows the clinical grouping of different empirical schemes based on meropenem (MEM) or cefoperazone/sulbactam (SCF). In 151 cases, the combination of two drugs was selected in 138 cases, 13 cases were treated with a combination of three drugs containing AZM. Of the 138 cases, 75 cases were based on MEM and 63 were based on SCF. In this retrospective study, the most commonly used antibiotic therapy for PA-VAP was MEM or SCF combined with AMK, AZM is rarely used in clinical treatment of *P. aeruginosa*. Table 4 shows the statistical analysis results of the clinical efficacy of different empirical treatment schemes.

**TABLE 3:** Clinical grouping of 151 cases.

	Antibiotic therapy (n)		n
Combination of two drugs (138)	Based on MEM (75)	MEM + AMK	34
		MEM + LEV	28
		MEM + AZM	13
	Based on SCF (63)	SCF + AMK	30
		SCF + LEV	23
		SCF + AZM	10
Combination of three drugs containing AZM (13)		MEM or SCF + AMK or LEV + AZM^a^	13

Abbreviations: MEM, meropenem; SCF, cefoperazone/sulbactam; AMK, amikacin; LEV, levofloxacin; AZM, azithromycin.

a There is no further subdivision of subgroups because of the small number of clinical cases.

**TABLE 4:** Therapeutic effect of different empirical schemes.

Antibiotic therapy (n)		Effective rate (%)	*P* value
Based on MEM (75)	MEM + AMK (34)	73.5 (25/34)	0.029^a^ * 0.173^b^ 0.768^c^
	MEM + LEV (28)	46.4 (13/28)	
	MEM + AZM (13)	69.2 (9/13)	
Based on SCF (63)	SCF + AMK (30)	70.0 (21/30)	0.025^d^ ^*^ 0.269^e^ 0.559^f^
	SCF + LEV (23)	39.1 (9/23)	
	SCF + AZM (10)	60.0 (6/10)	
Combined with AMK (64)	MEM + AMK (34)	73.5 (25/34)	0.754
	SCF + AMK (30)	70.0 (21/30)	
Combined with LEV (51)	MEM + LEV (28)	46.4 (13/28)	0.601
	SCF + LEV (23)	39.1 (9/23)	
Combined with AZM (23)	MEM + AZM (13)	69.2 (9/13)	0.645
	SCF + AZM (10)	60.0 (6/10)	
Combination of two or three drugs containing AZM (36)	MEM or SCF + AMK or LEV + AZM (13)	69.2 (9/13)	0.806
	MEM or SCF + AZM (23)	65.2 (15/23)	

Abbreviations: MEM, meropenem; SCF, cefoperazone/sulbactam; AMK, amikacin; LEV, levofloxacin; AZM: azithromycin.

a MEM + AMK vs. MEM + LEV.

b MEM + LEV vs. MEM + AZM.

c MEM + AKM vs. MEM + AZM.

d SCF + AMK vs. SCF + LEV.

e SCF + LEV vs. SCF + AZM.

f SCF + AKM vs. SCF + AZM.

* *p* < 0.05.

Among the 75 cases of two drug combination schemes based on MEM, 34 cases were MEM combined with AMK, and the effective rate was 73.5%; there were 28 cases of MEM combined with LEV, and the effective rate was 46.4%; 13 cases combined with AZM, and the effective rate was 69.2%. Intra group comparison found that the curative effect of MEM combined with AMK was significantly better than that of the LEV group, *p* = 0.029 (*p* < 0.05). There was no significant difference in the efficacy of MEM combined with LEV compared with MEM combined with AZM, at the same time, the efficacy of MEM combined with AMK was similar to that of MEM combined with AZM, with both *p* > 0.05.

Among the 63 cases of two drug combination schemes based on SCF, 30 cases were SCF combined with AMK, and the effective rate was 70%; there were 23 cases of SCF combined with LEV, and the effective rate was only 39.1%; 10 cases combined with AZM, and the effective rate was 60%. The efficacy of the combined LEV group was worse than that of the other two combinations. Further intra group comparison found that the curative effect of SCF combined with AMK was significantly better than that of the LEV group, *p* = 0.025 (*p* < 0.05). There was no significant difference in the efficacy of SCF combined with LEV compared with SCF combined with AZM, at the same time, the efficacy of SCF combined with AMK was similar to that of SCF combined with AZM, with both *p* > 0.05.

Among the two drug combination cases, 64 cases were combined with amikacin, 51 cases with levofloxacin, and 23 cases with azithromycin. In terms of effective rate alone, the effective rate of MEM combined with the aforementioned three drugs was higher than that of SCF. Further intra group statistical analysis found that there was no significant difference in the efficacy of MEM combined with AMK and SCF combined with AMK. The efficacy of LEV combined with MEM or SCF was similar, and there was no significant difference between MEM combined with AZM and SCF combined with AZM. All three *P* values were greater than 0.05.

Among 151 cases, 36 cases were combined with AZM, of which 23 cases were combined with two drugs and 13 cases were combined with three drugs. Because of the small number of cases, there was no further subgroup. It was found that there was no significant difference between combinations of two or three drugs containing AZM, *p* > 0.05 (*p* = 0.806). The details are shown in Table 4.

### Minimal Inhibitory Concentration Results of the *In Vitro* Drug Sensitivity Test

**FIGURE 2:**
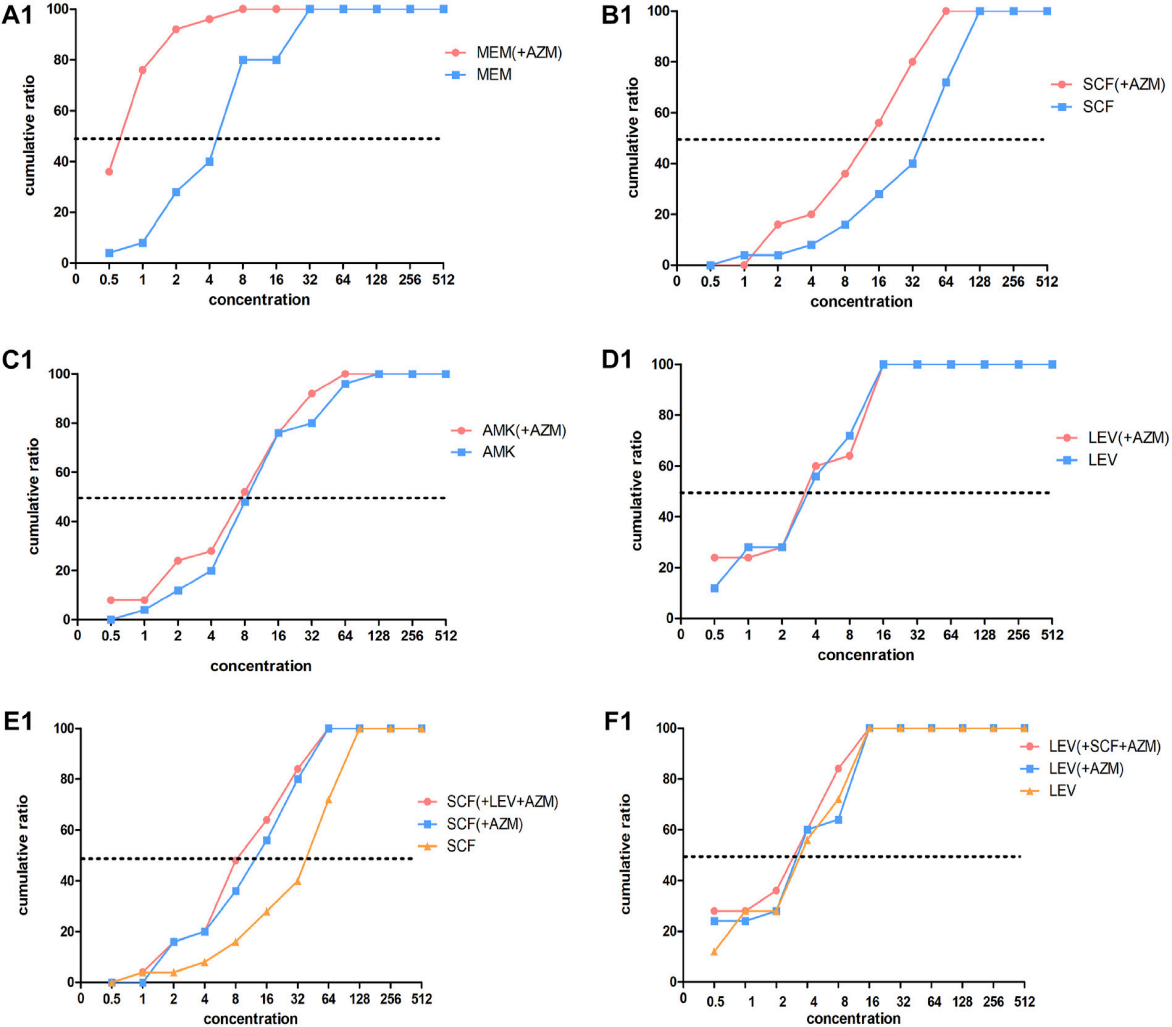
Concentration cumulative bacteriostatic percentage curves of four antibiotics. Abbreviations: MEM, meropenem; SCF, cefoperazone/sulbactam; AMK, amikacin; LEV, levofloxacin; AZM: azithromycin.

**FIGURE 3:**
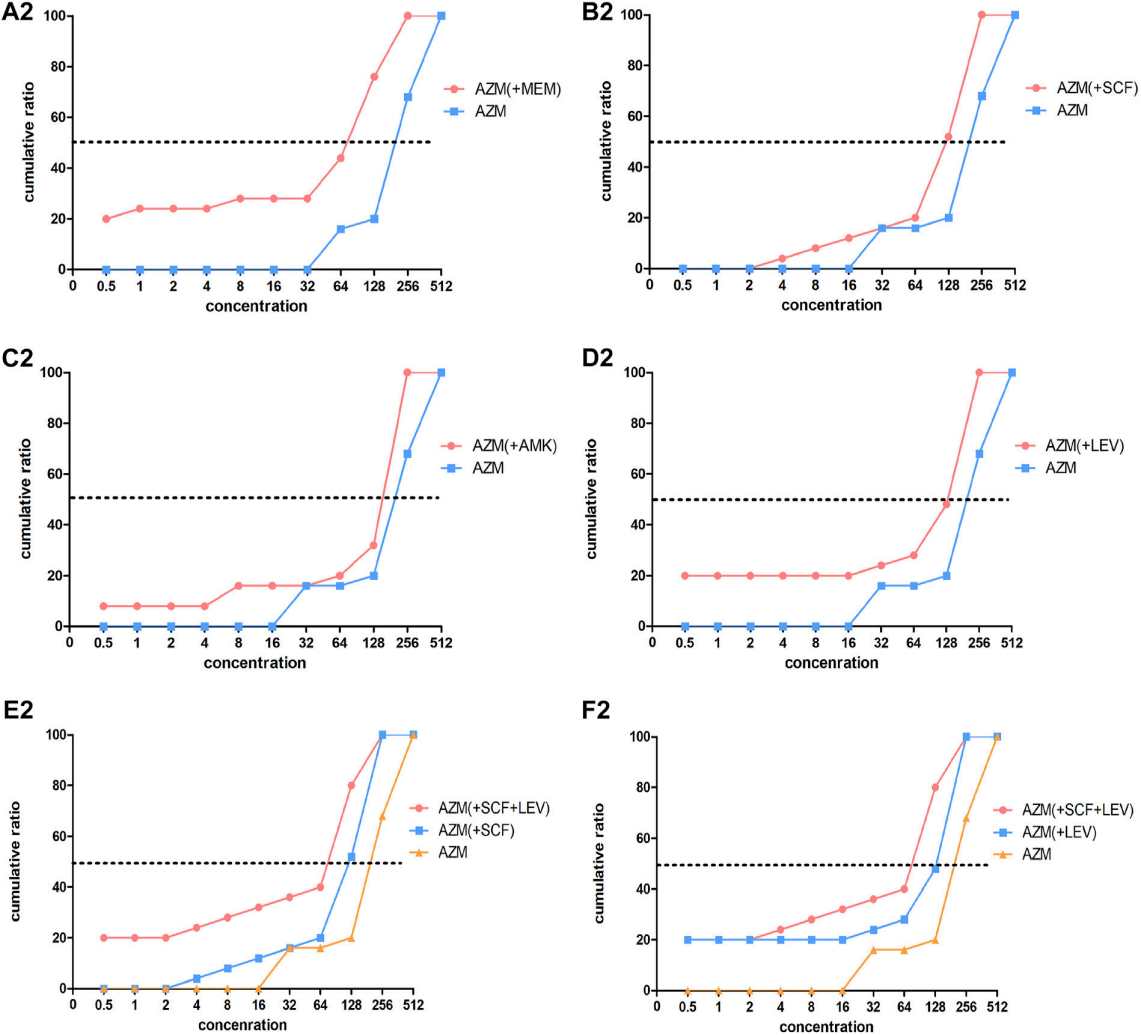
Concentration cumulative bacteriostatic percentage curves of azithromycin. Abbreviations: MEM, meropenem; SCF, cefoperazone/sulbactam; AMK, amikacin; LEV, levofloxacin; AZM: azithromycin.

A total of 27 strains and one quality control strain (ATCC 27853) were used for the *in vitro* drug sensitivity test. However, two strains were excluded from the analysis of the results due to failure in the experiment. Tables 5, 6, Figures 2, 3 show the MIC results of the five antibiotics against the 25 isolates of MDR-PA. Table 5 shows the MIC values of MEM, SCF, AMK, and LEV single drugs or after combined with AZM. Table 6 shows the MIC values of a AZM single drug or after combination. Figures 2, 3 show these percentage curves of concentration cumulative inhibition rate, respectively.

**TABLE 5:** MIC values of MEM, SCF, AMK, and LEV single drugs or after being combined with AZM against the 25 isolates of MDR *P. aeruginosa* (ug/mL).

Antibiotics	Alone			Combined with AZM			Combination of three drugs included AZM			Reference standard (CLSI-M100)		
	MIC_50_	MIC_90_	MIC_G_	MIC_50_	MIC_90_	MIC_G_	MIC_50_	MIC_90_	MIC_G_	S	I	R
MEM	8	32	0.5–32	1	2	0.5–16	—	—	—	≤2	4	≥8
SCF	64	128	1–128	16	64	2–64	8	64	2–64	≤16	32	≥64
AMK	4	16	0.5–16	4	16	0.5–16	—	—	—	≤16	32	≥64
LEV	8	64	1–128	8	32	0.5–64	4	16	0.5–16	≤2	4	≥8

Abbreviations: MIC, minimum inhibitory concentration; S, susceptible; I, intermediate; R, resistant.

**TABLE 6:** MIC values of the AZM single drug or after combination against the 25 isolates of MDR *P. aeruginosa* (ug/mL).

Antibiotic	MIC_50_	MIC_90_	MIC_G_	Reference standard
AZM alone	256	256	32–512	None
AZM + MEM	128	256	0.5–256	
AZM + SCF	128	256	4–256	
AZM + AMK	256	256	0.5–256	
AZM + LEV	128	256	0.5–256	
AZM (combination of three drugs)	128	256	0.5–256	

Abbreviations: MIC, minimum inhibitory concentration. S, susceptible; I, intermediate; R, resistant.

The MIC_50_ and MIC_90_ of a MEM single drug are 8 and 32 ug/mL, respectively. After being combined with AZM, MIC_50_ and MIC_90_ decreased to 1 and 2 ug/mL, respectively (which is reduced to the sensitivity critical point of MEM, i.e., ≤2 ug/mL). The MIC_50_ and MIC_90_ of the MEM decreased significantly and the concentration cumulative bacteriostatic percentage curve shifted significantly to the left (the inhibition effect was better) after being combined with AZM, as shown in Figure 2A1. The MIC_50_ and MIC_90_ of a SCF single drug are 64 and 128 ug/mL, respectively. After being combined with AZM, MIC_50_ decreased to 16 ug/mL, which is reduced to the sensitivity critical point of SCF, that is, ≤16 ug/mL, however, MIC_90_ decreased to 64, it was still in its resistant critical point, that is, ≥64 ug/mL. The concentration cumulative bacteriostatic percentage curve of SCF shifted obviously to the left after combined with AZM, as shown in Figure 2B1. The MIC_50_ and MIC_90_ of AMK single drug are 4 and 16 ug/mL, respectively (which were in sensitivity critical point of AMK, i.e., ≤16 ug/mL). After combined with AZM, MIC_50_ and MIC_90_ of AMK were same as single drug. The two curves of SCF were almost overlapping as shown in Figure 2C1. The MIC_50_ and MIC_90_ of LEV single drug were 8 and 64 ug/mL respectively. After being combined with AZM, MIC_50_ and MIC_90_ of LEV were 8 and 32 ug/mL, respectively. They were still at its resistance point, that is, ≥8. After being combined with AZM, the curve of LEV shifted implicitly, as shown in Figure 2D1. The MIC_50_ and MIC_90_ of AZM single drug were 256 ug/mL. After being combined with the other four drugs respectively, all MIC_50_ and MIC_90_ of AZM did not vary significantly. Four curves of AZM moved left in different degrees, as shown in Figures 3A2–D2.

To summarize, the AMK single drug was already very sensitive to the selected strains. When MEM or SCF was combined with AZM, the sensitivity of them to strains can be significantly increased; the sensitivity of LEV was improved after being combined with AZM, but it was not obvious.

Based on the aforementioned experimental results, we selected the scheme of SCF + LEV + AZM on the strains for further study. The analysis found that after the triple combination, the MIC_50_ and MIC_90_ of SCF and LEV were lower than those of the double combination, but they still could not achieve substantive changes. The MIC_90_ of SCF was reduced to 64, which was still in the range of the resistance level, that is, ≥64, and the MIC_50_ of LEV was reduced to 4, which still could not reach the sensitivity critical point, that is, ≤ 2. The left shift of SCF and LEV curves was not significant compared with the combination of three drugs and two drugs, as shown in Figures 2E1,F1. At the same time, after the combination of three drugs, the MIC_50_ and MIC_90_ of AZM were the same as those of the combination of two drugs, and the curve shift was slight, as shown in Figures 3E2,F2. The details are shown in Tables 5, 6.

### Fractional Inhibitory Concentration Index Results of the *In Vitro* Drug Sensitivity Test

The FIC index results of Table 7 suggested that, the main actions of MEM combined with AZM were the additive effect, accounting for 72%, the proportion of synergistic and additive effect added up to 80%, and 20% was the indifference effect; when SCF was combined with AZM, the addictive effect was 40%, and the unrelated effect was 60%; when LEV was combined with AZM, the additive effect accounted for 16%, and the unrelated effect accounted for 84%. After the combination of SCF + LEV + AZM, the additive effect accounted for 64%, and the unrelated effect accounted for 36% calculated based on SCF and AZM. In addition, the additive effect accounted for 32%, and the unrelated effect accounted for 68% analysis from the MIC of Lev and AZM. The details are shown in Table 7.

**TABLE 7:** Distribution (%) of the FIC index to the MDR *P. aeruginosa* (*n* = 25).

Antibiotic (n)		FIC ≤ 0.5	0.5 < FIC ≤ 1	1 < FIC ≤ 2	FIC>2
Combined with AZM	MEM	8 (2)	72 (18)	20 (5)	—
	SCF	—	40 (10)	60 (15)	—
	AMK	—	28 (7)	72 (18)	—
	LEV	—	16 (4)	84 (21)	—
SCF + LEV + AZM	SCF	—	64 (16)	36 (9)	—
	LEV	—	32 (8)	68 (17)	—

Abbreviations: FIC, fractional inhibitory concentration.

In conclusion, the combination of MEM and AZM showed the obvious additive effect. After the combination of SCF and AZM, the additive effect was 40%, and after the combination of three drugs, the additive effect was slightly increased to 64%. AMK or LEV combined with AZM mainly showed the unrelated effect, and the combination of three drugs could not improve the positive correlation between LEV and AZM.

## Discussion

The updated IDSA/ATS HAP/VAP guideline in 2016 specifically emphasizes that Hospital-acquired pneumonia (HAP) only refers to the pneumonia occurring after hospital admission in the patients without endotracheal intubation and is not associated with MV, while VAP represents the pneumonia occurring after endotracheal intubation and MV (Kalil et al., 2016). In China, people still assume that VAP is a special type of HAP (Shi et al., 2019). VAP is one of the most frequent ICU-acquired infections. Large scale studies worldwide have shown that the incidence of VAP is 2.5–40.0% (or 1.3 to 20.2 cases per 1,000 mechanical ventilation days) in ICU patients, associated with mortality of 13.0–25.2% (Kollef et al., 2012; Melsen et al., 2013). In our study, the incidence of VAP is approximately 10.4% (961/9245) of all mechanically ventilated patients in 5 years, consistent with the results of relevant studies (Melsen et al., 2013). VAP is associated with prolonged duration of MV and prolonged ICU stay (Papazian et al., 2020) and increased health-care costs (Zimlichman et al., 2013). Usual Gram-negative microorganisms involved in VAP are *P. aeruginosa*, *Escherichia coli*, *Klebsiella pneumoniae*, and *Acinetobacter* species; *Staphylococcus aureus* is the major Gram-positive microorganism (Bailey and Kalil, 2015; Huang et al., 2018; Luyt et al., 2018). A large proportion of VAP is caused by MDR pathogens and VAP in patients with risk factors for MDR pathogens is more likely to be due to MDR pathogens (Kalil et al., 2016). The non-standard use of antibiotics is one of the main factors for the occurrence of MDR pathogens.

MDR isolates of *P. aeruginosa* are increasingly prevalent (Denis et al., 2019). *P. aeruginosa* strains have recently become issues of public health concern (Oliver et al., 2015). One of three groups of antimicrobial-resistant Gram-negative bacteria posing particular therapeutic challenges is *P. aeruginosa* with difficult-to-treat resistance (DTR *P. aeruginosa*) according to the report of the US Centers for Disease Control and Prevention (CDC) antibiotic resistance threat (Thomas et al., 2007; Oliver et al., 2015). Recently, new tools using polymerase chain reaction (PCR) directly applied to fresh (bronchoscopic) samples have been developed to identify pathogens, which can shorten the time of organism identification and increased susceptibilities (Thomas et al., 2007). However, this technique is not available to determine *P. aeruginosa*. The prevalence of MDR *P. aeruginosa* is probably increasing worldwide, although with major geographical differences. The prevalence of MDR *P. aeruginosa* has increased over the last few decades and is now within the 15–30% range in multiple areas (Walkty et al., 2017; Sader et al., 2018).

Research showed *P. aeruginosa* had a high resistance to ciprofloxacin, levofloxacin, ceftazidime, piperacillin, imipenem, piperacillin and tazobactam, tobramycin, gentamicin, and meropenem, according to the data of a single center in Germany for 10 years (Yayan et al., 2015). Our statistical results of the resistance of *P. aeruginosa* to 14 antibiotics in 5 years showed that the resistants are furantoin (93.4%), aztreonam (62.9%), imipenem (50.3%), levofloxacin (47%), ciprofloxacin (46.4%), and meropenem (45%). It was worth mentioning that *P. aeruginosa* showed high resistance to carbapenem antibiotics from the clinical drug sensitivity results. The resistance rates of *P. aeruginosa* to imipenem and meropenem are 50.3% and 45.0%. In China, carbapenem-resistant *P. aeruginosa* (CR-PA) has been included in one of the five MDR bacteria targeted for prevention and control, in accordance with the requirements of the National Health Commission. During the last decade, there has been a global increase in the incidence and prevalence of carbapenem-resistant Gram-negative bacteria (Barbier and Luyt, 2016). In Europe, the population-weighted mean percentage of invasive isolates resistant to carbapenems in 2015 was 17.8% for *P. aeruginosa*. In the United States, 19.2% of *P. aeruginosa* submitted to the National Healthcare Safety Network was resistant to carbapenems in 2014 (Centers for Disease Control and Prevention, 2016; Tomczyk et al., 2019).

One of the main consequences of MDR is the difficulty of selecting an appropriate empirical antibiotic treatment. VAP caused by MDR bacteria puzzles every doctor in the ICU. Physicians face a dilemma, between avoiding ineffective treatment, inappropriate initial antimicrobial treatment being associated with increased mortality; and on the other hand, reducing the consumption of broad-spectrum antibiotics, the latter being associated with increased bacterial resistance (Yayan et al., 2015).

For patients with VAP due to *P. aeruginosa*, there are many choices according to the drug sensitivity results for sensitive *P. aeruginosa*, such as anti-PA cephalosporins and their combination with β-lactamase inhibitor complex preparations (such as ceftazidime and cefoperazone sulbactam), anti-PA carbapenems (including meropenem and biapenem), fluoroquinolones (ciprofloxacin and levofloxacin), aminoglycosides (amikacin and isopamicin), polymyxin, and fosfomycin based on clinical guidelines (Kalil et al., 2016; Torres et al., 2017; Shi et al., 2019). For patients with MDR-PA, the domestic and foreign guidelines for the treatment of PA-VAP recommend combination medication (ADSA-ATS in 2016 and Chinese guideline of 2018 Edition). For example, β-lactamase inhibitor compound preparation combined with fluoroquinolones or aminoglycosides, carbapenems combined with fluoroquinolones or aminoglycosides. For CR-PA, especially extremely drug-resistant (XDR) pulmonary infection, polymyxin (Kalil et al., 2016; Shi et al., 2019) and ceftazidime–avibactam are recommended (32–33).

Our study showed that the effective rate of MEM + AMK was 73.5% (25/34) and the SCF + AMK was 70% (21/30). The curative effect of MEM + AMK was better than that of the MEM + LEV group, *p* = 0.029 (*p* < 0.05) and this of SCF + AMK was better than that of the SCF + LEV group, *p* = 0.025 (*p* < 0.05). It indicated that the efficacy of MEM or SCF combined with AMK was better than that combined with LEV in the treatment of MDR-PA VAP. This can be explained by results of clinical drug sensitivity and *in vitro* drug sensitivity test. For 151 clinical cases, the sensitivity rate of amikacin was 88.1%, amikacin showed good sensitivity to most strains of *P. aeruginosa* (Pericolini et al., 2018). From the results of the drug sensitivity test *in vitro*, the MIC_50_ and MIC_90_ of AMK single drug are 4 and 16 ug/mL, respectively, which were in the sensitivity critical point of AMK, that is, ≤16 ug/mL. The high sensitivity of amikacin was further explained for *P. aeruginosa*. In contrast, the MIC_50_ and MIC_90_ of MEM, SCF, and LEV single drugs were in the range of drug resistance. American IDSA guidelines also pointed out that if DTR *P. aeruginosa* was not sensitive to all preferred drugs, a sensitive aminoglycoside can be considered in combination with cefloza–tazobactam, ceftazidime–avibactam, or imipenem–cilastatin–relibatan. The MIC closest to its sensitivity critical point was the preferred β-lactams β-lactamase inhibitor (Tamma et al., 2021). If DTR *P. aeruginosa* was also not sensitive to aminoglycosides, polymyxin B was considered in combination with preferred β-lactams β-lactamase inhibitor (Tamma et al., 2021).

However, amikacin alone is very rare because of its side reaction and the comprehensive situation of patients in ICU. In recent years, the application of polymyxin has gradually increased at home and abroad (Kalil et al., 2016; Vaara, 2019). However, compared with large provincial and teaching hospitals, the use in grass-roots hospitals is still very limited due to factors such as high costs or restrictions on prescription rights. Polymyxin was only available in our area last year. Also, a new β-lactams β-lactamase inhibitor (such as cefloza–tazobactam and ceftazidime–avibactam) is still in the clinical trial stage in China and has not been widely used in clinic (Papp-Wallace et al., 2019). Therefore, when the patient is with VAP caused by CR-PA, DTR-PA, or XDR *P. aeruginosa*, the treatment of VAP is more difficult for doctors in ICU. If the progress of the disease cannot be curbed in about a week, the function of other organs of the patient is bound to be affected, and serious secondary conditions such as septic shock and multiple organ failure (MOF) will develop.

In the ICU of our hospital, most patients with MDR *P. aeruginosa* VAP were treated with carbapenems (meropenem and biapenem) or commonly used β-lactamase inhibitor (cefoperazone sulbactam and piperacillin tazobactam) combined with aminoglycosides (amikacin and etimicin) or fluoroquinolones (moxifloxacin and levofloxacin). Based on the local drug supply at that time, the largest combination was meropenem or cefoperazone sulbactam combined with amikacin or levofloxacin. It is worth mentioning that a small number of patients were treated with the aforementioned drugs combined with azithromycin, possibly based on the medication situation at that time. Among the 75 cases of two drug combination scheme based on MEM, 13 cases combined with AZM, and the effective rate was 69.2% and the efficacy of MEM + AZM was similar to that of MEM + AMK, *p* > 0.05. Among the 63 cases of two-drug combination schemes based on SCF, 10 cases combined with AZM, and the effective rate was 60%. Also, the curative effect of SCF + AZM did not show significant difference in the efficacy of SCF + AMK, *p* > 0.05. We had surprisingly found that the combination of azithromycin has achieved a good therapeutic effect in the treatment of MDR *P. aeruginosa* VAP.

Azithromycin is the first broad-spectrum therapeutic drug, an azalide, a subclass of macrolide antibiotics. It prevents bacteria from growing by interfering with their protein synthesis. It binds to the 50S subunit of the bacterial ribosome and thus inhibits translation of mRNA (Bakheit et al., 2014). Azithromycin is used to treat or prevent certain bacterial infections, most often those causing middle ear infections, strep throat, typhoid, bronchitis, and sinusitis. Azithromycin (AZM) was used to treat chronic inflammatory airway diseases because it regulates the cell–cell contact between airway epithelial cells. AZM can inhibit the ability of TNF-α-to induce interleukin (IL)-8 production (Yang, 2020). In fact, azithromycin is not often selected by doctors in ICU. However, in the Strategic Plan for Biodefense Research by the US Department of Health and Human Services, azithromycin is one such broad-spectrum therapeutic that is both included in the University of Oxford’s RECOVERY and excluded from the World Health Organization’s SOLIDARITY trialsI. The Strategic Plan will demarcate the need for drugs which target multiple types of pathogens to prepare for infectious threat (Firth and Prathapan, 2020). The latest research has shown that azithromycin was used to treat COVID-19 (Damle et al., 2020; Oldenburg et al., 2021).

The treatment options for nosocomial Gram-negative infections are very limited. The poor activities of these antibiotics on bacterial biofilms and the increasing prevalence of MDR *P. aeruginosa* leave the physicians with very limited choices to effectively treat these patients (Bassetti et al., 2018; Ciofu and Tolker Nielsen, 2019; Kumar et al., 2019). Antibiotic-resistant biofilms exist widely in *P. aeruginosa* infection, which is one of the important reasons for the failure of antibacterial treatment. Biofilm is an architecture built mostly by autogenic extracellular polymeric substances which functions as a scaffold to encase the bacteria together on surfaces, and to protect them from environmental stresses, impeding phagocytosis and thereby conferring the capacity for colonization and long-term persistence (Mah et al., 2003; Sharma et al., 2014; Kang and Kirienko, 2018; Thi et al., 2020). Macrolide antibiotics have little activity against *P. aeruginosa*. However, it can inhibit the formation of biofilm, regulate immunity, enhance the phagocytosis of phagocytes, and inhibit some toxic factors of *P. aeruginosa*. Macrolide antibiotics may enhance the antibacterial activity of other drugs against *P. aeruginosa* by destroying the biofilm and improving the curative effect. The results of Ren et al. (2019) indicated that the trans-translation system played an essential role in *P. aeruginosa* tolerance to azithromycin and multiple aminoglycoside antibiotics which was a ribosome rescue system that plays an important role in bacterial tolerance to environmental stresses. The experimental result showed that the ciprofloxacin–azithromycin sinus stent (CASS) maintained a uniform coating and sustained delivery of ciprofloxacin and azithromycin, providing anti-biofilm activities against *P aeruginosa* (Lim et al., 2020). Raouf et al. (2021) indicated that combined free and ciprofloxacin–azithromycin nanoparticles on chitosan nanocarrier (Cipro-AZM-CS) showed promising results *in vitro* and *in vivo* overcoming high resistance of biofilm producing *P. aeruginosa*. The present study was conducted to evaluate the effect of azithromycin on *P. aeruginosa* biofilm. We showed that azithromycin exhibited a potent activity against *P. aeruginosa* biofilm, and microscopic observation revealed that azithromycin substantially inhibited the formation of solid surface biofilms. Interestingly, we observed that azithromycin restricted the *P. aeruginosa* biofilm formation by inhibiting the expression of pel genes. We concluded that azithromycin attenuates *P. aeruginosa* biofilm formation, impairs its ability to produce the extracellular biofilm matrix, and increases its sensitivity to the immune system (Kumar et al., 2021).

From the MIC results of our vitro drug sensitivity test, we found that after MEM + AZM, MIC_50_ and MIC_90_ of MEM reduced the sensitivity critical point of MEM, that is, ≤2 ug/mL from 8 to 32 ug/mL; after SCF + AZM, MIC_50_ of SCF decreased to 16 ug/mL, which is reduced to the sensitivity critical point of SCF, that is, ≤16 ug/mL. The concentration cumulative bacteriostatic percentage curve of MEM shifted significantly to the left. The FIC index results suggested that the main actions of MEM combined with AZM were the additive effect, accounting for 72%, and the proportion of synergistic and additive effect is added up to 80%. The proportion of the additive effect of SCF + AZM was 40%. We speculated that azithromycin may increase the bioactivity of meropenem and cefoperazone sulbactam by destroying the biofilm of multidrug-resistant *Pseudomonas aeruginosa*. This effect is particularly obvious after combining meropenem from our experimental results. The underlying mechanism related to this will be further studied in our future research. At the same time, we also found that the combination of three drugs containing azithromycin is not necessary to treatment of MDR *P. aeruginosa* ventilator-associated pneumonia, whether from the clinical results or *in vitro* drug sensitivity test results.

## Conclusion

Our study suggested that MDR *P. aeruginosa* was highly sensitive to amikacin in our region. Carbapenems or cephalosporins-β-lactamase compound combined with amikacin had a good effect in the treatment of VAP of MDR *P. aeruginosa*. At the same time, azithromycin was combined with carbapenems or cephalosporins-β-lactamase compound could to be selected as the recommended scheme. In primary hospitals, we recommend azithromycin to treat MDR *P. aeruginosa* VAP when amikacin is resistant and polymyxin or ceftazidime–avibactam is not available. Moreover, the second is enough, and the third is unnecessary, which cannot further increase the therapeutic effect.

## Data Availability

The original contributions presented in the study are included in the article/Supplementary Material; further inquiries can be directed to the corresponding authors.

## References

[B1] Al-Orphaly M. Hadi H. A. Eltayeb F. K. Al-Hail H. Samuel B. G. Sultan A. A. (2021). Epidemiology of Multidrug-Resistant *Pseudomonas Aeruginosa* in the Middle East and North Africa Region. mSphere 6 (3), e00202–21. 10.1128/mSphere.00202-21 34011686 PMC8265635

[B2] Bailey K. L. Kalil A. C. (2015). Ventilator-Associated Pneumonia (VAP) with Multidrug-Resistant (MDR) Pathogens: Optimal Treatment? Curr. Infect. Dis. Rep. 17, 494. 10.1007/s11908-015-0494-5 26092246

[B3] Bakheit A. H. Al-Hadiya B. M. Abd-Elgalil A. A. (2014). Azithromycin. Profiles Drug Subst. Excip. Relat. Methodol. 39, 1–40. 10.1016/B978-0-12-800173-8.00001-5 24794904

[B4] Barbier F. Luyt C. E. (2016). Understanding Resistance. Intensive Care Med. 42, 2080–2083. 10.1007/s00134-016-4543-9 27647333

[B5] Bassetti M. Vena A. Croxatto A. Righi E. Guery B. (2018). How to Manage *Pseudomonas Aeruginosa* Infections. Drugs Context 7, 212527. 10.7573/dic.212527 29872449 PMC5978525

[B6] Centers for Disease Control and Prevention (2016). Antibiotic Resistance Patient Safety Atlas 2016. Atlanta: Centers for Disease Control and Prevention.

[B7] Centers for Disease Control and Prevention (2019). Antibiotic Resistance Threats in the United States. Georgia: CDC.

[B8] Ciofu O. Tolker Nielsen T. (2019). Tolerance and Resistance of *Pseudomonas Aeruginosa* Biofifilms to Antimicrobial Agents How *P. Aeruginosa* Can Escape Antibiotics. Front. Microbiol. 10, 913. 10.3389/fmicb.2019.00913 31130925 PMC6509751

[B9] Clinical and Laboratory Standards Institute (2022). Performance Standards for Antimicrobial Susceptibility Testing. 32nd Edition. Malvern: CLSI.

[B10] Daikos G. L. da Cunha C. A. Rossolini G. M. Stone G. G. Baillon-Plot N. Tawadrous M. (2021). Review of Ceftazidime-Avibactam for the Treatment of Infections Caused by *Pseudomonas Aeruginosa*. Antibiotics. Basel) 10 (9), 1126. 10.3390/antibiotics10091126 PMC8467554 34572708

[B11] Damle B. Vourvahis M. Wang E. Leaney J. Corrigan B. (2020). Clinical Pharmacology Perspectives on the Antiviral Activity of Azithromycin and Use in COVID-19. Clin. Pharmacol. Ther. 108 (2), 201–211. 10.1002/cpt.1857 32302411 PMC7262099

[B12] Denis J. B. Lehingue S. Pauly V. Cassir N. Gainnier M. Leone M. (2019). Multidrug-resistant *Pseudomonas aeruginosa* and Mortality in Mechanically Ventilated ICU Patients. Am. J. Infect. Control 47, 1059–1064. 10.1016/j.ajic.2019.02.030 30962023

[B13] Faure E. Kwong K. Nguyen D. (2018). *Pseudomonas aeruginosa* in Chronic Lung Infections:how to Adapt within the Host[J]. Front. Immunol. 9, 2416. 10.3389/fimmu.2018.02416 30405616 PMC6204374

[B14] Firth A. Prathapan P. (2020). Azithromycin: The First Broad-Spectrum Therapeutic. Eur. J. Med. Chem. 207, 112739. Epub 2020 Aug 19. 10.1016/j.ejmech.2020.112739 32871342 PMC7434625

[B15] Huang Y. Jiao Y. Zhang J. Xu J. Cheng Q. Li Y. (2018). Infection Assembly of Shanghai Respiratory SMicrobial Etiology and Prognostic Factors of Ventilator-Associated Pneumonia: a Multicenter Retrospective Study in Shanghai. Clin. Infect. Dis. 67, S146–S152. 10.1093/cid/ciy686 30423049

[B16] Kalanuria A. Zai W. Mirski M. (2014). Ventilator-associated Pneumonia in the ICU. Crit. Care 18 (2), 208. 10.1186/cc13775 25029020 PMC4056625

[B17] Kalil A. C. Metersky M. L. Klompas M. Muscedere J. Sweeney D. A. Palmer L. B. (2016). Executive Summary: Management of Adults with Hospital-Acquired and Ventilator-Associated Pneumonia: 2016 Clinical Practice Guidelines by the Infectious Diseases Society of America and the American Thoracic Society. Clin. Infect. Dis. 63 (5), 575–582. 10.1093/cid/ciw504 27521441 PMC4981763

[B18] Kang D. Kirienko N. V. (2018). Interdependence between Iron Acquisition and Biofilm Formation in *Pseudomonas Aeruginosa* . J. Microbiol. 56 (7), 449–457. 10.1007/s12275-018-8114-3 29948830 PMC6221862

[B19] Kollef M. H. Hamilton C. W. Ernst F. R. (2012). Economic Impact of Ventilator-Associated Pneumonia in a Large Matched Cohort. Infect. Control Hosp. Epidemiol. 33 (3), 250–256. 10.1086/664049 22314062

[B20] Kumar M. Rao M. Mathur T. Barman T. K. Joshi V. Chaira T. (2021). Azithromycin Exhibits Activity against *Pseudomonas Aeruginosa* in Chronic Rat Lung Infection Model. Front. Microbiol. 12, 603151. 10.3389/fmicb.2021.603151 33967970 PMC8102702

[B21] Kumar M. Rao M. Purnapatre K. P. Barman T. K. Joshi V. Kashyap A. (2019). DS86760016, a Leucyl tRNA Synthetase Inhibitor with Activity against *Pseudomonas Aeruginosa* . Antimicrob. Agents Chemother., 63, e02122–18. 10.1128/AAC.02122-18 30670430 PMC6437482

[B22] Liao S. Zhang Y. Pan X. Zhu F. Jiang C. Liu Q. (2019). Antibacterial Activity and Mechanism of Silver Nanoparticles against Multidrug-Resistant *Pseudomonas Aeruginosa* . Int. J. Nanomedicine 14, 1469–1487. 10.2147/IJN.S191340 30880959 PMC6396885

[B23] Lim D. J. Skinner D. Mclemore J. Rivers N. Elder J. B. Allen M. (2020). *In-Vitro* Evaluation of a Ciprofloxacin and Azithromycin Sinus Stent for *Pseudomonas Aeruginosa* Biofilms. Int. Forum Allergy Rhinol. 10 (1), 121–127. 10.1002/alr.22475 31692289 PMC6942221

[B24] Luyt C. E. Hekimian G. Koulenti D. Chastre J. (2018). Microbial Cause of ICU-Acquired Pneumonia: Hospital-Acquired Pneumonia Versus Ventilator-Associated Pneumonia. Curr. Opin. Crit. Care 24, 332–338. 10.1097/MCC.0000000000000526 30036192

[B25] Mah T. F. Pitts B. Pellock B. Walker G. C. Stewart P. S. O'Toole G. A. (2003). A Genetic Basis for *Pseudomonas Aeruginosa* Biofilm Antibiotic Resistance. Nature 426 (6964), 306–310. 10.1038/nature02122 14628055

[B26] Maurice N. M. Bedi B. Sadikot R. T. (2018). Pseudomonas aeruginosaBiofilms: Host Response and Clinical Implications in Lung Infections. Am. J. Respir. Cell. Mol. Biol. 58 (4), 428–439. 10.1165/rcmb.2017-0321TR 29372812 PMC5894500

[B27] Melsen W. G. Rovers M. M. Groenwold R. H. Camus C. Bauer T. T. Hanisch E. W. (2013). Attributable Mortality of Ventilator-Associated Pneumonia: A Meta-Analysis of Individual Patient Data from Randomised Prevention Studies. Lancet Infect. Dis. 13 (8), 665–671. 10.1016/S1473-3099(13)70081 23622939

[B28] Metersky M. L. Kalil A. C. (2018). Management of Ventilator-Associated Pneumonia, Clin. Chest Med. 39 (4), 797–808. 10.1016/j.ccm.2018.08.002 30390750

[B29] Mills J. P. Marchaim D. (2021). Multidrug-Resistant Gram-Negative Bacteria. Infect. Dis. Clin. N. Am. 35 (4), 969–994. 10.1016/j.idc.2021.08.001 34752228

[B30] Miyoshi-Akiyama T. Tada T. Ohmagari N. Viet Hung N. Tharavichitkul P. Pokhrel B. M. (2017). Emergence and Spread of Epidemic Multidrug-Resistant *Pseudomonas Aeruginosa* . Genome Biol. Evol. 9 (12), 3238–3245. 10.1093/gbe/evx243 29202180 PMC5726472

[B31] Oldenburg C. E. Pinsky B. A. Brogdon J. Chen C. Ruder K. Zhong L. (2021). Effect of Oral Azithromycin vs Placebo on COVID-19 Symptoms in Outpatients with SARS-CoV-2 Infection. A Randomized Clin. Trial. JAMA 326 (6), 490–498. 10.1001/jama.2021.11517 PMC8285753 34269813

[B32] Oliver A. Mulet X. López-Causapé C. Juan C. (2015). The Increasing Threat of *Pseudomonas Aeruginosa* High-Risk Clones. Drug Resist. Updat. 21–22, 41–59. 10.1016/j.drup.2015.08.002 26304792

[B33] Otsuka Y. (2020). Potent Antibiotics Active against Multidrug-Resistant Gram-Negative Bacteria. Chem. Pharm. Bull. 68 (3), 182–190. 10.1248/cpb.c19-00842 32115524

[B34] Papazian L. Klompas M. Luyt C.-E. (2020). Ventilator-Associated Pneumonia in Adults: A Narrative Review. Intensive Care Med. 46 (5), 888–906. Epub 2020 Mar 10. 10.1007/s00134-020-05980-0 32157357 PMC7095206

[B35] Papp-Wallace K. M. Zeiser E. T. Becka S. A. Park S. Wilson B. M. Winkler M. L. (2019). Ceftazidime-Avibactam in Combination with Fosfomycin: A Novel Therapeutic Strategy against Multidrug-Resistant *Pseudomonas Aeruginosa* . J. Infect. Dis. 220 (4), 666–676. 10.1093/infdis/jiz149 31099835 PMC6639593

[B36] Pericolini E. Colombari B. Ferretti G. Iseppi R. Ardizzoni A. Girardis M. (2018). Real-Time Monitoring of *Pseudomonas Aeruginosa* Biofilm Formation on Endotracheal Tubes Vitro. BMC Microbiol. 18 (1), 84. 10.1186/s12866-018-1224-6 30107778 PMC6092828

[B37] Raouf M. Essa S. El Achy S. Essawy M. Rafik S. Baddour M. (2021). Evaluation of Combined Ciprofloxacin and Azithromycin Free and Nano Formulations to Control Biofilm Producing *Pseudomonas Aeruginosa* Isolated from Burn Wounds. Indian. J. Med. Microbiol. 39 (1), 81–87. 10.1016/j.ijmmb.2021.01.004 33460732

[B38] Ren H. Liu Y. Zhou J. Long Y. Liu C. Xia B. (2019). Combination of Azithromycin and Gentamicin for Efficient Treatment of *Pseudomonas Aeruginosa* Infections. J. Infect. Dis. 220 (10), 1667–1678. 10.1093/infdis/jiz341 31419286

[B39] Ribeiro Á. C. D. S. Crozatti M. T. L. Silva A. A. D. Macedo R. S. Machado A. M. D. O. Silva A. T. D. A. (2019). *Pseudomonas Aeruginosa* in the ICU: Prevalence, Resistance Profile, and Antimicrobial Consumption. Rev. Soc. Bras. Med. Trop. 53, e20180498. 10.1590/0037-8682-0498-2018 31859938 PMC7083346

[B40] Sader H. S. Castanheira M. Duncan L. R. Flamm R. K. (2018). Antimicrobial Susceptibility of Enterobacteriaceae and *Pseudomonas Aeruginosa* Isolates from United States Medical Centers Stratified by Infection Type: Results from the International Network for Optimal Resistance Monitor- Ing (INFORM) Surveillance Program. Diagn Microbiol. Infect. Dis. 92, 69–74. 10.1016/j.diagmicrobio.2018.04.012 29789189

[B41] Sharma G. Rao S. Bansal A. Dang S. Gupta S. Gabrani R. (2014). *Pseudomonas Aeruginosa* Biofilm: Potential Therapeutic Targets. Biologicals 42 (1), 1–7. 10.1016/j.biologicals.2013.11.001 24309094

[B42] Shi Y. HuangY. Zhang T. T. Cao B. Wang H. Zhuo C. (2019). Chinese Guidelines for the Diagnosis and Treatment of Hospitalacquired Pneumonia and Ventilator-Associated Pneumonia in Adults (2018 Edition). J. Thorac. Dis. 11 (6), 2581–2616. 10.21037/jtd.2019.06.09 31372297 PMC6626807

[B43] Souza G. H. A. Rossato L. Brito G. T. Bet G. M. D. S. Simionatto S. (2021). Carbapenem-Resistant *Pseudomonas Aeruginosa* Strains: a Worrying Health Problem in Intensive Care Units. Rev. Inst. Med. Trop. Sao Paulo 63, e71. 10.1590/S1678-9946202163071 34586305 PMC8494492

[B44] Tamma P. D. Aitken S. L. Bonomo R. A. Mathers A. J. van Duin D. Clancy C. J. (2021). Infectious Diseases Society of America Guidance on the Treatment of Extended-Spectrum-Lactamase Producing Enterobacterales (ESBL-E),Carbapenem-Resistant Enterobacterales (CRE),and *Pseudomonas Aeruginosa* with Difficult-To-Treat Resistance (DTR-P. Aeruginosa). Clin. Infect. Dis. 72 (7), e169–e183. PMID: 33106864. 10.1093/cid/ciaa1478 33106864

[B45] Thi M. T. T. Wibowo D. Rehm B. H. A. (2020). *Pseudomonas Aeruginosa* Biofilms. Int. J. Mol. Sci. 21 (22), 8671. 10.3390/ijms21228671 PMC7698413 33212950

[B46] Thomas L. C. Gidding H. F. Ginn A. N. Olma T. Iredell J. (2007). Development of a Real-Time *Staphylococcus Aureus* and MRSA (SAM-) PCR for Routine Blood Culture. J. Microbiol. Methods 68, 296–302. 10.1016/j.mimet.2006.09.003 17046087

[B47] Tomczyk S. Zanichelli V. Grayson M. L. Anthony T. Abbas M. Pires D. (2019). Control of Carbapenem-Resistant Enterobacteriaceae, Acinetobacter Baumannii, and *Pseudomonas Aeruginosa* in Healthcare Facilities: A Systematic Review and Reanalysis of Quasi-Experimental Studies. Clin. Infect. Dis. 68 (5), 873–884. 10.1093/cid/ciy752 30475989 PMC6389314

[B48] Torres A. Niederman M. S. Chastre J. Ewig S. Patricia F. V. Hanberger H. (2017). International ERS/ESICM/ESCMID/ALAT Guidelines for the Management of Hospital Acquired Pneumonia and Ventilator-Associated Pneumonia: Guidelines for the Management of Hospital Acquired Pneumonia (HAP)/Ventilator-Associated Pneumonia (VAP)of the European Respiratory Society (ERS),European Society of Intensive Care Medicine (ESICM),European Society of Clinical Microbiology and Infectious Diseases (ESCMID)and Asociacion Latinoamericana del Torax (ALAT). Eur. Respir. J. 50 (3), 1700582. 10.1183/13993003.00582-2017 28890434

[B49] Vaara M. (2019). Polymyxin Derivatives that Sensitize Gram-Negative Bacteria to Other Antibiotics. Molecules 24 (2), 249. 10.3390/molecules24020249 PMC6359160 30641878

[B50] Walkty A. Lagace-Wiens P. Adam H. Baxter M. Karlowsky J. Mulvey M. R. (2017). Antimicrobial Susceptibility of 2906 *Pseudomonas Aeruginosa* Clinical Isolates Obtained from Patients in Canadian Hospitals Over a Period of 8 years: Results of the Canadian Ward Surveillance Study (CANWARD), 2008-2015. Diagn Microbiol. Infect. Dis. 87, 60–63. 10.1016/j.diagmicrobio.2016.10.003 28336136

[B51] Yang J. (2020). Mechanism of Azithromycin in Airway Diseases. J. Int. Med. Res. 48 (6), 300060520932104. 10.1177/0300060520932104 32589092 PMC7323306

[B52] Yayan J. Ghebremedhin B. Rasche K. Yayan J. Ghebremedhin B. Rasche K. (2015). Antibiotic Resistance of *Pseudomonas Aeruginosa* in Pneumonia at a Single University Hospital Center in Germany over a 10-Year Period. PLoS One 10, e0139836. 10.1371/journal.pone.0139836 26430738 PMC4592231

[B53] Zimlichman E. Henderson D. Tamir O. Franz C. Song P. Yamin C. K. (2013). Health Care-Associated Infections: A Meta-Analysis of Costs and Fnancial Impact on the US Health Care System. JAMA Intern. Med. 173, 2039–2046. 10.1001/jamainternmed.2013.9763 23999949

